# Effects of COVID-19 pandemic on pediatric weight: A retrospective chart review

**DOI:** 10.1016/j.pmedr.2022.102109

**Published:** 2023-01-02

**Authors:** Kelly M. Dopke, Krista L. Pattison, Eric W. Schaefer, Benjamin N. Fogel, Deepa L. Sekhar

**Affiliations:** a Pediatrics, Penn State College of Medicine, Hershey, PA, United States; b Public Health Sciences, Penn State College of Medicine, Hershey, PA, United States

**Keywords:** Pediatrics, Weight gain, Healthcare disparities, COVID-19, BMI, Body mass index, SD, standard deviation, IQR, interquartile range, CI, confidence interval

## Abstract

The COVID-19 pandemic forced United States school closures in March 2020. Students moved to online learning, fostering a sedentary lifestyle. As the pandemic heightened population disparities, the impact on weight gain may also be unequally distributed. This study aimed to evaluate changes in body mass index (BMI) z-scores and weight percentiles of pediatric patients during the pandemic and associated demographics to identify those at risk for weight gain. Methods included a retrospective chart review of patients 5–18 years-old with a well-visit in the three years 2018, 2019 and 2020; first identified with a well-visit in August-September of 2020. BMI z-scores and weight percentiles were analyzed using a correlated errors regression model appropriate for longitudinal data. This longitudinal approach was used to model outcomes by patient demographics. Interaction terms with time were evaluated for each variable. Of 728 patients, mean age was 9.7 years (2018); 47 % female, 70 % white, and 23 % publicly insured. BMI z-score did not increase significantly from 2018-2019 versus 2019–2020. Weight percentile demonstrated a slight trajectory increase over these same time points. Publicly insured patients demonstrated significantly greater increase in BMI z-score versus privately insured patients (p = 0.009). Mean differences between groups increased from 0.26 in 2018 (95 % CI [0.07, 0.45]) to 0.42 in 2020 (95 % CI [0.23, 0.61]). Results were similar for weight percentile. Publicly insured pediatric patients experienced significant increase in BMI-z score and weight percentile, but over time this trajectory remained constant. The results support targeting at risk subgroups in addressing long-term impacts of the pandemic.

## 1 Introduction

In mid-March 2020, Pennsylvania schools closed in response to the COVID-19 pandemic. Similar to school districts nationwide, this resulted in students transitioning to online learning environments via mobile devices and computers, fostering a more sedentary lifestyle (Ammar et al., 2020). The increased time spent at home raised concerns about sedentary lifestyle, screen usage, and reduction in organized time for physical activity (Zachary et al., 2020). United States schools struggled incorporating physical activity while limiting potential COVID-19 transmission (Springboard to active schools, 2020).

Prior work on the impact of the pandemic reported decreased physical activity, specifically among adolescents, and perceived weight gain (Elnaggar et al., 2020, Di Renzo et al., 2020). Woolford et al. quantified this gain with Kaiser Permanente data, estimating a gain of 2.30 kg more based on body mass index (BMI) measured June 2020-January 2021 versus pre-pandemic (March 2019-January 2020) for 5–11-year-olds, 2.31 kg for 12–15-year-olds, and 1.03 kg for 16–17-year-olds (Woolford et al., 2021).

The pandemic is well-documented to have heightened existing disparities in the United States population. Thus, the impact on weight gain may also be unequally distributed. Our academic outpatient pediatrics providers anecdotally noted weight fluctuations during the pandemic, most often weight gain, among children presenting for well-child visits. Yet, the extent of these weight changes and their association with specific demographic factors is unknown. To better understand weight gain for our patients during the pandemic, especially disparities in weight gain, we used available data from the Penn State Health pediatric outpatient practices to compare changes in BMI z-score and weight percentiles between school age pediatric patients from 2018 through the COVID-19 pandemic in 2020. We evaluated associations between weight changes and basic demographic factors (sex, race, ethnicity, and insurance status).

## 2 Methods

This retrospective chart review used electronic health record data from four pediatric outpatient practice sites of an academic medical center. For inclusion patients had to be school age, born between August 31, 2002 (18 years in 2020) and August 31, 2013 (5 years in 2018), have a well-visit August 1 to September 30, 2020, and have prior well-visits recorded in 2019 and 2018 for comparison. For each of the three well-visits available information had to include at least one of the following: BMI value, BMI percentile, BMI z-score and weight value (kilograms) and weight percentile on the Centers for Disease Control and Prevention pediatric growth curves.  This study was approved by the Penn State College of Medicine Institutional Review Board.

Demographic data was summarized. We analyzed BMI z-scores and weight percentiles over time using a linear regression model (correlated errors) for longitudinal data. The same framework was used for all models, with the only change being the specific variables included in the models. The variance–covariance matrix was specified as unstructured. Missing values for the outcome were excluded (18 for BMI z-score [0.8 %] and 10 for weight percentile [0.5 %]); no other variables were missing data.

We began by fitting a model that included only year as a covariate (with 2018 the reference). The parameters of the model indicated the mean values for each year. Based on model parameters, we compared the mean change from 2018 to 2019 to the mean change from 2019 to 2020 to investigate whether the COVID-19 pandemic affected the trajectory over time.

Next, we fit models that included year (as above) plus the following patient characteristics: sex (male/female), baseline age in 2020 (≤8 years-old, >8 to ≤ 11 years old, >11 to ≤ 13 years old, and > 13 to ≤ 16 years old), race (non-Hispanic/other), ethnicity (white/other), and insurance (public or private determined in 2020) with uninsured patients categorized as public. We evaluated interactions of each variable with time. The only significant interaction term (time and insurance status) was retained. As in the model that included only time, we compared the difference in mean changes (2019 to 2020 versus 2018 to 2019) for public insurance to the same difference in mean changes for private insurance using the estimated model parameters. Thus, the difference in trajectory over time for private insurance was compared to the difference in trajectory over time for public insurance (a difference-in-difference approach).

## 3 Results

728 patients met inclusion criteria. Mean age was 9.7 years (2018); patients were 47 % female, 70 % white, 80 % non-Hispanic and 23 % publicly insured. Mean BMI z-scores were 0.36 (standard deviation [SD] = 1.09) in 2018, 0.42 (SD = 1.07) in 2019, and 0.47 (SD = 1.09) in 2020. Mean weight percentiles were 58.5 (SD = 29.6) in 2018, 59.8 (SD = 29.9) in 2019, and 62.1 (SD = 29.3) in 2020. Median follow up from 2018 to 2019 was 372 days (interquartile range [IQR] 368 to 385 days), and median follow up from 2019 to 2020 was 375 days (IQR 370 to 408 days).

Parameter estimates were obtained from the linear regression models for BMI z-score and weight percentile with year as the only factor. Compared to 2018, mean BMI z-scores were 0.06 higher in 2019 (95 % confidence interval [CI] [0.03, 0.09]) and 0.13 higher in 2020 (95 % CI [0.09, 0.17]). Thus, mean BMI z-scores were 0.07 higher in 2020 versus 2019 (95 % CI [0.04, 0.11]). Comparing the change from 2018 to 2019 (mean increase of 0.06) to the change from 2019 to 2020 (mean increase of 0.07) is a difference of 0.01, which was not statistically significant (95 % CI [-0.03, 0.06]; p = 0.46).

Similarly, compared to 2018, mean weight percentile was 1.2 points higher in 2019 (95 % CI [0.6, 1.9]) and 3.6 points higher in 2020 (95 % CI [2.5, 4.6]). Thus, mean weight percentile was 2.4 points higher in 2020 compared to 2019 (95 % CI [1.6, 3.2]). Comparing the change from 2018 to 2019 (mean increase of 1.2) to the change from 2019 to 2020 (mean increase of 2.4) is a difference of 1.2, which was statistically significant (95 % CI [0.2, 2.2]; p = 0.023).

Table 1 shows parameter estimates from the linear regression models for BMI z-score and weight percentile that contained year and demographic factors. A statistically significant interaction was observed between insurance and year (p = 0.009) such that mean BMI z-score increased more rapidly for publicly insured compared to privately insured patients. Specifically, the difference in mean BMI z-scores between groups (public versus private insurance) increased from 0.26 in 2018 (95 % CI [0.07, 0.45]) to 0.42 in 2020 (95 % CI [0.23, 0.61]). Year-to-year mean BMI z-score changes were 0.03 and 0.04 for private insurance and 0.10 and 0.13 for public insurance, for 2018 to 2019 and 2019 to 2020, respectively. Comparing these yearly mean changes (difference of 0.03 [0.13–0.10] for public insurance versus difference of 0.01 [0.04–0.03] for private insurance) yields a difference of 0.02, which was not statistically significant (95 % CI [−0.09, 0.13]; p = 0.72).

**Table 1:** Patient characteristics and parameter estimates from fitted model for BMI z-score and weight percentile.

BMI z-score			
Parameter	Estimate (SE)	95 % CI	p-value
Intercept	0.34 (0.11)	(0.13, 0.55)	0.002
Year, private insurance group (reference group for interaction)			
2018 (ref)	0		
2019	0.03 (0.02)	(−0.01, 0.07)	0.09
2020	0.07 (0.03)	(0.02, 0.13)	0.008
Age			
≤8 years old (ref)	0		
>8 to ≤ 11 years old	0.04 (0.03)	(−0.03, 0.11)	0.26
>11 to ≤ 13 years old	0.06 (0.05)	(−0.03, 0.16)	0.20
>13 years old	0.09 (0.06)	(−0.03, 0.21)	0.15
Sex			
Male	0.03 (0.08)	(−0.12, 0.19)	0.65
Female (ref)	0		
Race			
White	−0.02 (0.11)	(−0.23, 0.19)	0.85
Other (ref)	0		
Ethnicity			
Non-Hispanic	−0.10 (0.12)	(−0.33, 0.14)	0.43
Other (ref)	0		

**Weight percentile**			
Intercept	55.8 (2.9)	(50.0, 61.5)	<0.001
Year, private insurance group (reference group for interaction)			
2018 (ref)	0		
2019	0.5 (0.4)	(−0.3, 1.3)	0.20
2020	2.0 (0.7)	(0.7, 3.3)	0.003
Age			
≤8 years old (ref)	0		
>8 to ≤ 11 years old	1.5 (0.8)	(−0.1, 3.0)	0.07
>11 to ≤ 13 years old	2.5 (1.2)	(0.2, 4.7)	0.034
>13 years old	4.2 (1.5)	(1.4, 7.1)	0.004
Sex			
Male	2.4 (2.1)	(−1.8, 6.5)	0.26
Female (ref)	0		
Race			
White	−0.6 (2.9)	(−6.3, 5.1)	0.82
Other (ref)	0		
Ethnicity			
Non-Hispanic	−1.0 (3.3)	(−7.5, 5.6)	0.77
Other (ref)	0		

A statistically significant interaction was also observed between insurance and year (p = 0.032) for weight percentile. Specifically, the difference in mean weight percentiles between groups (public versus private insurance) increased from 4.8 points in 2018 (95 % CI [−0.4, 10.0]) to 8.1 points in 2020 (95 % CI [2.9, 13.2]). Year-to-year mean weight percentile changes were 0.5 and 1.4 points for private insurance and 1.8 and 3.4 points for public insurance. Comparing these yearly mean changes (difference of 1.6 [3.4–1.8] for public insurance versus difference of 0.9 [1.4–0.5] for private insurance) yields a difference of 0.7, which was not statistically significant (95 % CI [−1.7, 3.1]; p = 0.56).

**Fig. 1:**
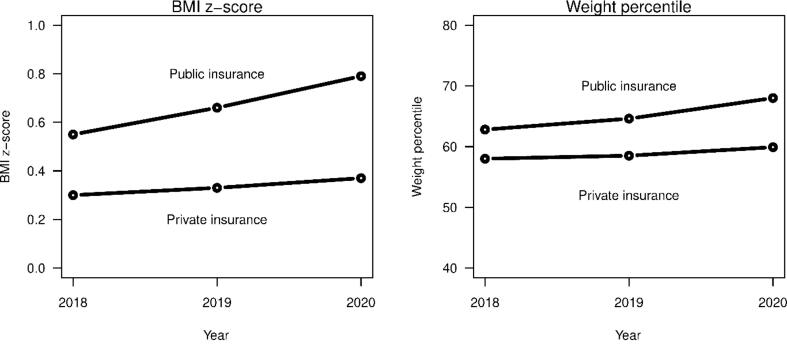
Model-based estimates of mean BMI z-scores and weight percentiles for publicly and privately insured pediatric patients. All other model parameters were set to the most common category.

Fig. 1 shows the mean estimates over time by insurance groups from the models for each outcome. Age was the only other demographic factor significant in the model for weight percentile, demonstrating that older patients had larger mean weight percentiles.

## 4 Discussion

In this study of change in BMI z-score and weight percentiles of pediatric patients during the COVID-19 pandemic, public insurance was significantly associated with increased BMI z-score and weight percentiles. This finding supports published data on disparities in pediatric weight gain.

Childhood obesity is one of our nation’s greatest public health challenges. Prior research on school attendance and weight gain demonstrates that obesity related risk factors are more pronounced during school breaks. This is because during these times children are exposed to ‘gaining weight environments’ (Kang et al., 2021). Specifically, during school breaks, reduction in physical activity and an increase in unhealthy dietary habits, compounded by irregular sleep schedules and longer screen time exposure may exacerbate weight gain and decrease cardiopulmonary fitness (Wang et al., 2020). In some ways, the shelter in place orders during the COVID-19 pandemic mirrored the weight gaining environments described during school breaks. However, social interaction was even more limited during quarantine, placing the pediatric population at greater risk for adverse effects on physical and mental health (Ye, 2020). These adverse effects were further perpetrated by disparate access to care during COVID-19 (Wang et al., 2020). Our findings are consistent with the current literature on pediatric weight gain and the impact of social determinants of health on disparities in weight gain among at-risk youth from low-income families (Brooks et al., 2021, Eneli et al., 2022).

Multiple studies report varying increases in BMI z-score during the COVID-19 pandemic among pediatric patients (Woolford et al., 2021, Kang et al., 2021, Brooks et al., 2021, Beck et al., 2022). Kang et al. determined that the duration of school closure was significantly associated with increase in BMI (p = 0.004) (Kang et al., 2021). Weaver et al.’s analysis of change in BMI z-score during COVID-19 found an increase of 0.33 (95 % CI [0.16, 0.50]) with greater changes among children who were not overweight or obese at baseline (Weaver et al., 2021). Woolford et al. found that youth during the pandemic gained more weight than pre-pandemic with an absolute increase of overweight and obesity of 5.2 % among 12–15-year-olds (Woolford et al., 2021). These findings collectively suggest greater increases in pediatric weight gain during the pandemic. Additional work supports that weight gain was most pronounced among already vulnerable groups (Brooks et al., 2021, Beck et al., 2022).

Brooks et al’s study of weight gain during COVID-19 included a population in which 15.7 % of children had public insurance or were uninsured. Results demonstrated weight gain was most prominent among children with pre-existing obesity, Hispanic children, and children who lacked commercial or private insurance (Brooks et al., 2021). Specifically, the overall increase in mean BMI was 0.43 (95 % CI [0.37, 0.50]) greater in 2020 when compared to previous years for those that were publicly insured or without insurance (Brooks et al., 2021). Similarly in Jenssen et al’s study, when looking at the outcome of obesity, preexisting disparities in insurance, race and socioeconomic status widened as a result of the pandemic (Jenssen et al., 2021).

Our findings demonstrate patients with public insurance had an accelerated trajectory of weight gain compared to their privately insured counterparts. Though this trajectory was not significantly accelerated during the COVID-19 pandemic, the findings highlight an unequal impact of insurance status on weight gain. The aforementioned findings provide insight on areas for health prevention targeting at risk populations. For primary care providers, the findings highlight the need to be mindful of disparities in counseling families on weight gain. Providers may consider focusing on inexpensive ways to encourage physical activity/healthy food habits (e.g. walking to school, online exercise video) and opening the conversation to problem-solve practical solutions with families. Additionally, providers could consider working with their local school districts to develop physical activity plans (e.g. the creation of student health teams) as a nationwide survey highlighted schools’ prioritization of the physical environment upon the re-entry to in-person instruction as a primary focus with a secondary focus on physical and mental health of students and staff (Pattison et al., 2021).

## 5 Study limitations and strengths

The study is limited in including data from a single academic institution. Thus, the patient population may be a limiting factor in detecting interactions. The time frame was three years and data on well-visits from August to September 2020 were approximately six-months into the pandemic in the United States. Thus, the post-COVID period may have been too short to observe changes. In addition, there may be selection bias in terms of who was willing to come to the office for a well-visit during this time. Despite these limitations the results are timely, relevant, and support the growing body of literature on disparities in pediatric weight.

## 6 Conclusion

Publicly insured pediatric patients experienced a more accelerated increase in BMI z-score and weight percentile during the pandemic than privately insured counterparts, though this trajectory was not significantly accelerated during the pandemic. The results confirm existing literature documenting health disparities and weight gain, though this was not associated with the pandemic based on our results.

## 7 Disclosure of funding

Funding for the open access publication fees was received, in part, through Children's Miracle Network.

## CRediT authorship contribution statement

**Kelly M. Dopke:** Conceptualization, Validation, Investigation, Writing – original draft, Writing – review & editing, Visualization. **Krista L. Pattison:** Conceptualization, Investigation, Writing – original draft, Writing – review & editing, Visualization. **Eric W. Schaefer:** Methodology, Software, Writing – original draft, Writing – review & editing, Visualization. **Benjamin N. Fogel:** Conceptualization, Methodology, Supervision, Project administration, Writing – review & editing. **Deepa L. Sekhar:** Conceptualization, Methodology, Validation, Investigation, Writing – review & editing, Visualization, Supervision, Funding acquisition, Project administration.

## Declaration of Competing Interest

The authors declare that they have no known competing financial interests or personal relationships that could have appeared to influence the work reported in this paper.

## Data Availability

The data that has been used is confidential.
